# PDL-1 Antibody Drug Conjugate for Selective Chemo-Guided Immune Modulation of Cancer

**DOI:** 10.3390/cancers11020232

**Published:** 2019-02-16

**Authors:** Samaresh Sau, Alex Petrovici, Hashem O. Alsaab, Ketki Bhise, Arun K. Iyer

**Affiliations:** 1 Department of Pharmaceutical Sciences, Wayne State University Eugene Applebaum College of Pharmacy and Health Sciences, 259 Mack Ave, Detroit, MI 48201, USA; alex.o.petrovici@gmail.com (A.P.); hashem.alsaab@wayne.edu (H.O.A.); ketki.bhise@wayne.edu (K.B.); 2 Department of Pharmaceutics and Pharmaceutical Technology, College of Pharmacy, Taif University, Taif 26571, Saudi Arabia; 3 Molecular Imaging Program, Barbara Ann Karmanos Cancer Institute. Wayne State University School of Medicine, 4100 John R St, Detroit, MI 48201, USA

**Keywords:** antibody drug conjugate (ADC), PD-L1, tumor spheroid disruption, immune modulation, doxorubicin

## Abstract

Targeting immune checkpoint molecules such as programmed death ligand-1 (PDL1) is an emerging strategy for anti-cancer therapy. However, transient expression of PDL1 and difficulty in tumor stroma penetration has limited the utility of anti-PDL1 therapy. To overcome these limitations, we report a new conjugate between the clinically approved PDL1 antibody (PDL1 AB) and drug Doxorubicin (Dox), named PDL1-Dox. We conjugated PDL1-Dox through a hydrazone linker containing a polyethylene glycol (PEG) spacer, which allows it to dissociate in a tumor environment and improves solubility. The purpose of using Dox is to disrupt the tumor extracellular environment so that PDL-1 antibody can penetrate the tumor core. PDL1-Dox demonstrates significant cell killing, disruption of tumor spheroid and induction of apoptosis in a breast cancer cell line. Significant release of IFN-γ suggests PDL1-Dox can upmodulate T cell activation. Optical imaging of dye conjugate supports the selective tumor targeting ability and core penetration of the construct.

## 1. Introduction

Antibody-drug conjugates (ADCs) are a clinically effective treatment for targeted therapy of cancer. They typically consist of a monoclonal antibody, a cytotoxic drug, and a conditionally stable linker to conjugate the two. In cancer treatment, such combinations are especially useful because the antibody (Ab) serves as a specific targeting ligand to an overexpressed tumor cell surface protein in order to effectively deliver the cytotoxic drug [1]. So far, three ADCs (Adcetris, Kadcyla and Mylotarg) have been approved by the US FDA and more than 30 ADCs are currently being investigated in clinical trials for both solid tumors and hematological cancers [2]. Recently, groundbreaking results of immunotherapy have opened a new paradigm for several cancer treatments [3]. A promising target in anticancer therapy is immune checkpoint inhibition which resurrects the function of exhausted T-cells to kill tumor cells. Tumor cells evade immune surveillance by upmodulating immunosuppressive immune checkpoint molecules, resulting in downplay of antitumor immunity [3,4]. This involves the interaction between the surface receptor programmed death-1 (PD1) and its corresponding ligand (PDL1), which are expressed on the surface of immune cells (monocytes, T cells, B cells) and tumor cells, respectively [3]. The interaction between PD1 of T-cells with PDL1 of cancer cells inhibits T-cell mediated cancer cell killing. To alleviate the function of T-cells against cancer cells several immune checkpoint antibody inhibitors have been developed that target either PD1 or PDL1 and stop this interaction.

Tecentriq® (Atezolizumab), an FDA approved antibody, has already been used for metastatic urothelial carcinoma, non-small cell lung cancer, and triple negative breast cancer (TNBC) [5]. Hypothetically, this antibody can be used against other types of tumors that have overexpression of PDL1. Several studies have shown that TNBC cells, including MDA-MB-231, have high expression of PDL1 [6], which suggests that an anti-PDL1 antibody is a promising platform for TNBC therapy [4]. Since TNBC cells lack receptors for estrogen, progesterone, and HER-2 [7], using the PDL1 biomarker is a rational option for its treatment. Alongside its clinical success, the treatment of anti-PDL1 antibody showed a patient specific response. However, its use is limited to the few types of tumors that are linked with several factors, such as the transient and heterogeneous expression of PDL1 in tumor microenvironment and poor penetration of the larger molecular weight PDL1 antibody (144.61 Kda) through dense tumor stroma [8,9]. Thus, an attempt to conjugate a cytotoxic drug with anti-PDL1 antibody would be a significant direction especially for solid tumors consisting of dense tumor stroma. In this regard, PDL1 antibody drug conjugate (ADC) can serve the purpose of chemo-guided immune therapy.

Chemotherapeutics such as Doxorubicin (Dox) have been utilized as potent anticancer agents for a long time. They work by slowing the growth of cancer cells through induction of apoptosis and arresting cell cycle that leads to cell death [10,11]. The poor selectivity and acute cardiotoxicity of Dox has limited its use in clinic, requiring a selective delivery system. Clinical use of Doxil is widespread and the predicted market size is expected to be $1.39 billion by 2024 [12]. Alongside this, a few antibody-Dox conjugates, including BR96-Dox (NCT00031187) and PL1-Dox (NCT01101594), have been studied in clinical trials for cancer [13]. These data support the idea that there is a significant scope in repurposing Dox for efficient therapy in cancer. Toward this end, we report for the first time a Dox conjugated PDL1 antibody (PDL1-Dox) for the broader application of chemo-guided immunotherapy.

**Figure 1:**
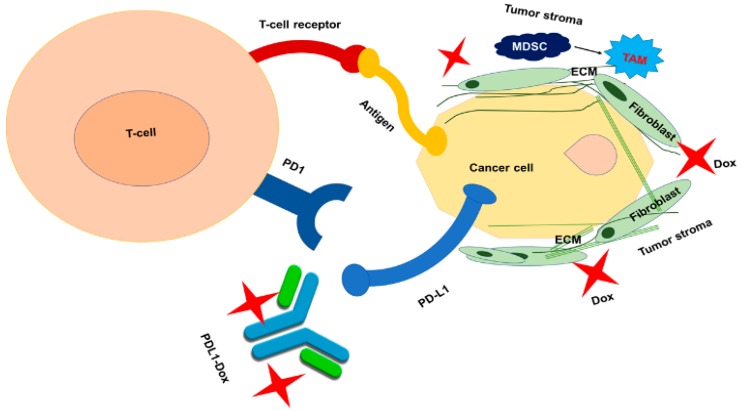
The interaction of the cancer cell with the T-cell through the binding of the major histocompatibility complex (MHC) and the T-cell receptor (TCR) leads to activation of the T-cell and releasing cancer cell death signal. The ligation of PD1 with PDL1 downmodulates the tumor cell killing function of T-cell. It is hypothesized that Dox of PDL1-Dox could disrupt the tumor stromal components and improve antitumor response of PDL1 antibody.

As the tumor extracellular environment is acidic in nature, acidic pH responsive linkers have been utilized to conjugate ADCs so that they can selectively release drugs (Dox in this case) in the tumor environment [14]. Likewise, we have introduced a hydrazone linker to the PDL1-Dox ADC that will be selectively cleaved in the tumor cell environment. Additionally, we used a PEG-spacer for improving the aqueous solubility of the antibody and sustain the plasma circulation of PDL1-Dox. The hydrazone linker is extensively used for clinically approved ADCs such as Mylotarg. The monoclonal IgG1 antibody, PDL1 AB, has a high affinity for human PDL1 receptor with a dissociation constant (Kd) of 0.43 nM [15]. PDL1 AB binds to the PDL1 on the surface of the cancer cell and it does not internalize via endocytosis, resulting in inhibition of PDL1 with PD1 of T cells. Alongside the superior clinical outcome of PDL1 AB, several studies have revealed that its effect is limited to the small percent of patient population [16]. This is attributed to poor T cell infiltration through dense tumor stroma [17] and inadequate tumor core penetration of PDL1 AB, as depicted in Figure 1. To overcome these challenges, several combination therapies have emerged, including combination of PDL1 AB with chemotherapy and immunotherapies, namely anti-PD1 or CTLA-4 therapy [3,4,5]. Combination treatment, however, produced nonspecific toxicity and immune related adverse events (irAEs) [18,19]. To improve the selectivity and efficacy of PDL1 AB, we have developed the ADC, PDL1-Dox and evaluated its anticancer effect and mechanism of action in MDA-MB-231 cells. The chemical formation of PDL1-Dox was confirmed by MALDI-MS spectroscopy and UV-Vis spectrophotometry. We performed a tumor 3D- culture study to demonstrate the tumor spheroid disruption ability of PDL1-Dox and measured IFN-γ production in PDL1-Dox treated cell suspension, obtained from a co-culture of MDA-MB-231 and activated RAW 264.7 cells. We developed near-infrared (NIR) dye-conjugated PDL1-S0456 and tested its specificity as well as tumor retention ability in patient derived TNBC (BR1126) and NSCLC (LG703) model. The tumor specificity of PDL1-S0456 was confirmed by ex-vivo biodistribution on treated PDx mice. This proof-of-concept study demonstrates that PDL1-Dox can improve the current therapeutic outcome beyond PDL1 AB and that PDL1 antibody can further be developed for tumor diagnosis and image-guided surgery [20].

## 2. Materials and Methods

### 2.1. Materials

Atezolizumab (Tecentriq®, Genentech, South San Francisco, CA, USA) was obtained from Karmanos Cancer Institute pharmacy. SH-PEG-COOH was obtained from Biochempeg Scientific Inc., (Watertown, MA, USA). Other reagents and solvents were obtained from fisher scientific and Sigma Aldrich and used directly without further purification. RAW 264.7 cells were obtained as a kind gift of Shunbin Xu, Wayne State University School of Medicine (Detroit, MI, USA).

### 2.2. Synthesis of PDL1-Dox

The synthesis of the ADC began with the coupling of the monoclonal IgG antibody Atezolizumab (Tecentriq®, Genentech) to SH-PEG-COOH by EDC/sulfo-NHS, according to previously published method [21]. Clinically used PDL1 AB was dialyzed to separate the excipient and PDL1 AB was lyophilized to obtain the powered. For coupling between SH-PEG-COOH and PDL1 AB, SH-PEG-COOH (15 mg) was taken in a in mixture of water with catalytic amount DMSO in presence of EDC/sulfo-NHS and stirred for 1 h, followed by PDL1 AB powder (18 mg) was added to the mixture and stirred for 6 h. The resulting solution was transferred to a 12 Kd dialysis bag and dialyzed overnight at 4 °C to obtain PDL1-PEG-SH. Next PDL1-PEG-SH was then reacted with maleimide group of Aldoxorubicin in pH 7.4 using reagent free thiol-maleimide chemistry for 4 h. Followed by dialysis and lyophilization was performed with 12 Kd dialysis bag to obtain PDL1-Dox. The concentration of Dox was determined by the UV-Vis spectroscopy method.

### 2.3. Characterization of PDL1-Dox

PDL1-Dox was analyzed in UV/Vis spectroscopy (Agilent Technologies, Santa Clara, CA, USA) to evaluate the presence of Dox in the PDL1-Dox construct and compared with PDL1 AB.

### 2.4. Cell Culture

TNBC cell line (MDA-MB-231) was obtained from American Type Culture Collection (ATCC, Manassas, VA, USA) and grown in with Dulbecco’s modified eagle medium, containing 1% antibiotic (penicillin and streptomycin) and 10% fetal bovine serum at 37 °C in a 5% CO_2_ environment. MTT based cell viability assay was performed as per previously performed procedures [5,22,23]. Briefly, the cells were seeded in a 96 well plate at a density of 5000 cells per well and incubated overnight. Afterwards, the cells were treated with various concentrations of PDL1-DOX in a range of 2.5 μM to 0.156 μM with respect to Dox concentration and cells were incubated for 48 h or 72 h. The same amount of commercial Dox was used as a positive control. At the end of incubation, 3-(3,5-dimethyl-2-thiazolyl)-2,5-diphenyltetrazolium bromide (MTT) was added and cell viability was determined. Standard manufacturer procedure was followed for 3D-spheroid culture method. Briefly, 5000 MDA-MB-231 cells were slowly added to U-shaped well of 96-well plate and incubated for overnight. This was followed by PDL1-Dox, with Dox being treated for 48 h or kept untreated.

### 2.5. Apoptosis Assay

In preparation for the apoptosis assay, cells were seeded in 6-well plates for 24 hours. The cells were then treated with PDL1-DOX or kept untreated (UT) at concentrations of 2.5 μM and then incubated for 24 h, until a microscopically visible morphology change was occurred. The cells were then collected, centrifuged, counted, resuspended, and analyzed with a guava Guava® easyCyte™ flow cytometer (Austin, TX, USA). 

### 2.6. Cellular Imaging with Confocal Microscopy

Confocal imaging was performed based on previously published literature [22]. Briefly, MDA-MB-231 cells were plated with a density of 200,000 cells per 60 mm petri dish and waited 48 h until confluency reached up to 70%, then cells were treated with 1 μM concentration of PDL1-Dox or Dox with respect to Dox concertation for 1 h in 10% FBS containing DMEM. The cells that followed were washed 3 times with PBS and fixed with 2% formalin in PBS for 15 min. 15 min prior to confocal imaging, cells were stained with Hoechst 33342. Dox was visualized in red channel (Ex. 488 nm and Em. 560 nm) and Hoechst was visualized in blue channel (Ex. 350 nm, Em. 461 nm) and images were merged to demonstrate the localization of PDL1-Dox and Dox in cells [11].

### 2.7. 3D-spheroid Culture Study

5,000 MDA-MB231 cells/well were plated in 3D-matrix containing 96-well plate and waited for 18 h to form 3D-spehroid. Following this, spheroids were treated with 5 μM and 2.5 μM of Dox and PDL1-Dox with respect to Dox concentration for 20 h and bight field images were taken in a phase contrast microscope under 4× objective.

### 2.8. IFN-γ ELISA

The enzyme linked immunosorbent assay (ELISA) of the IFN-γ cytokine was performed with bioligand ELISA kit as per manufacturer protocol. Briefly, 5000 RAW 264.7 cells/well were seeded to 96-well plate for 18 h. In day 2, media was changed with 1 μg/mL LPS containing DMEM for 24 h. On Day 3, 4000 MDA-MB-231 cells/well were co-cultured with RAW 264.7 cells containing wells in presence of 1 μg/mL LPS. Day 4, cells were treated with Dox (2.5 μM), PDL1-Dox (2.5 μM) or left UT for 24 h. Day 5, media was collected and run for Elisa assay. The quantity of IFN-γ was quantified with the IFN-γ standard.

### 2.9. Animal Studies

All animal procedures and imaging experiments was done according to protocols approved by the Institutional Laboratory Animal Care & Use Committee (IACUC) at the Wayne State University. Near-infrared (NIR) dye, S0456 was conjugated with PDL1-PEG-SH to obtain PDL1-S0456 and free S0456 was separated by dialysis [7]. The 8-weeks old tumor bearing TNBC and NSCLC patient derived tumor xenograft (PDx) mice were intravenously injected with 10 nmole of PDL1-S0456 TNBC and the bio-distribution of NIR dye was monitored after 24 h of the single dose of 10 nmole NIR dye per mouse. Non-specific BSA-S0456 was used as a control. Fluorescence images were collected in Bruker Carestream Xtreme in vivo imaging system at excitation (750 nm) and emission (830 nm) wavelength as per previously published method [7]. The instrument has dual fluorescence and X-ray imaging modalities with light source and fluorescence and X-ray images of the mouse were merged to demonstrate the localization of NIR dye. PDx tumor mice were obtained from Jackson laboratory, and tumor fragments were passaged to Nod-Scid mice.

### 2.10. Statistical Analysis

The statistical analysis was done using GraphPad Prism 7 software (GraphPad Software Inc., La Jolla, CA, USA). The data were expressed as mean ± SD and analyzed using a two-tailed Student *t*-test, or one-way ANOVA followed by a post hoc test. A *p*-value of <0.05 was considered statistically significant.

## 3. Results

### 3.1. Synthesis and Characterization

**Figure 2:**
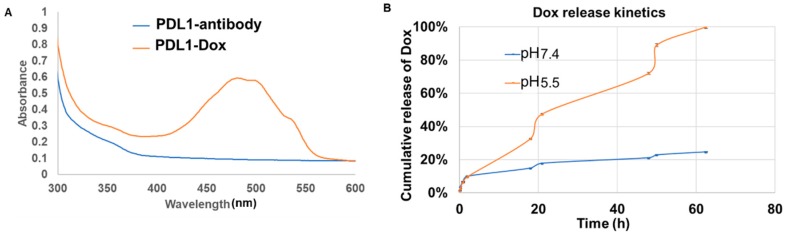
(**A**) The presence of the Dox absorbance peak in PDL1-Dox indicates successful conjugation of Dox. (**B**) The Dox is more completely released from the conjugate in more acidic conditions due to hydrazone linker degradation.

The synthesis of the ADC was performed similar to previously published literature using the clinically used PDL1 antibody, COOH-PEG-SH and Aldoxorubicin [24]. The PDL1 AB itself does not have any significant absorbance at the same wavelength of max absorbance of Doxorubicin (481 nm) as can be found in Figure 2A. Therefore, the intensity of the absorbance at 481 nm was used to determine the concentration of Dox present in the PDL1-Dox formulation. This concentration of Dox in PDL1-Dox was considered as the basis for performing biological studies of PDL1-Dox, as it could be compared to free Dox. This is justified because the difference between in vitro activity of PDL1-Dox compared to free Dox could be attributed to the presence of PDL1 AB. The data from Figure 2A demonstrate the successful conjugation of Dox with PDL1 AB through PEG linker to produce PDL1-Dox ADC. As mentioned, the use of hydrazone linker in PDL1-Dox is needed to selectively deliver Dox to the extracellular acidic milieu of the tumor so that it can disrupt the tumor environment and enhance the penetration of PDL1-antibody into the core of the tumor [1]. To demonstrate the acidic pH responsive release of Dox from PDL1-Dox, we studied the release kinetics of PDL1-Dox in PBS of pH 5.5 and pH 7.4. Figure 2B indicates that 90% of Dox was released in pH 5.5 at 50 h, whereas the released amount of Dox was less than 30% in pH 7.5 at 50 h. The sustained and acidic pH stimuli-responsive release of Dox from PDL1-Dox supports the hypothesis of using PDL1-Dox ADC for chemo-guided immunotherapy in preclinical model.

### 3.2. Cell Killing

**Figure 3:**
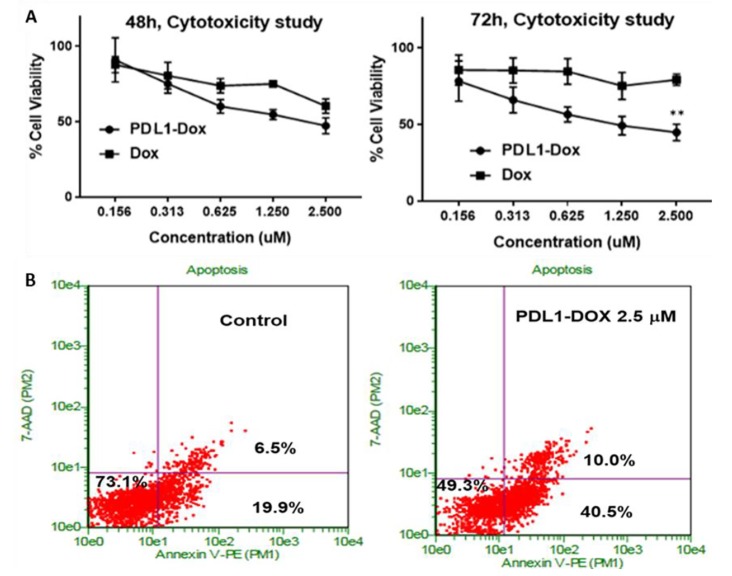
(**A**) The MTT based cell viability assay in MDA-MB-231 indicates the PDL1-Dox is more effective in killing PDL-1 overexpressing MDA-MB-231 cells as compared to Dox (*n* = 6). (**B**) The cell killing of PDL1-Dox is mediated by early apoptosis pathway. ** *p* < 0.01.

Figure 3A shows the MTT-based cytotoxicity assay in MDA-MB-231 cells that displayed a dose dependent cell killing of PDL1-Dox treatment. The difference of cell killing of PDL1-Dox and Dox is more prominent at 72 h as compared to 48 h, indicating time dependent cell killing effect of PDL1-Dox in PDL1 overexpressing MDA-MB-231 cells [6]. The cell killing effect of PDL1-Dox is significantly higher in the range of 0.625 µM to 2.5 µM as compared to Dox treated cells for 72 h treatment. The reason for the superior cell killing effect of PDL1-Dox in the lower concentrations can be attributed to PDL-1 receptor mediated and acidic pH triggered Dox release. The IC50 of Dox and PDL1-Dox is 4 µM and 1.25 µM respectively. Thus, the cell viability data indicate conjugation of Dox with PDL-1 antibody significantly improved the cell killing effect of Dox at 72 h that corroborated with sustain drug release kinetics data obtained in acidic pH, as shown in Figure 2B. This observation supports the notion that PDL1-Dox will function as a potent tumor environment specific Dox delivery agent. To demonstrate the cell killing mechanism of PDL1-Dox, we performed Annexin-V/PE based apoptosis assay and the data is shown in Figure 3B. The results indicate a significant increase in early phase apoptosis in PDL1-Dox in comparison to untreated control (UT). The percent of early stage apoptosis in PDL1-Dox treated MDA-MB-231 is 2-fold higher compared to UT cells, suggesting that PDL1-Dox is highly efficient in inducing apoptosis-mediated cell death. With this efficient anticancer effect of PDL1-Dox, we sought to explore the cross-talk mechanism of PDL1-Dox with the MDA-MB-231 cells. The PDL1 AB binds with extracellularly overexpressed PDL1 receptor of cancer cells, resulting in the inhibition of interaction between PDL1 with PD-1 of T cell and the induction of T cell mediated tumor cell killing.

### 3.3. Cell Uptake

**Figure 4:**
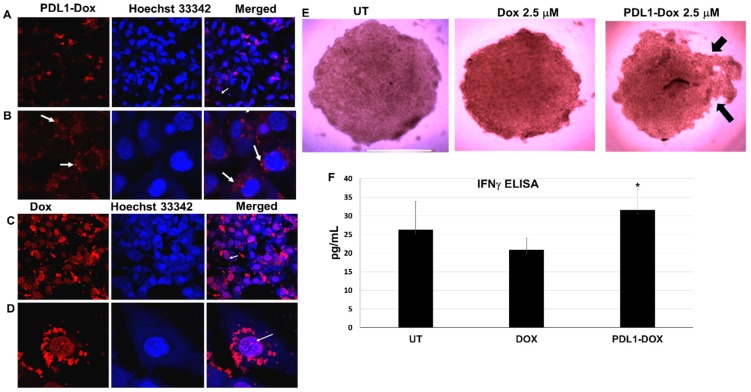
(**A**) Cell uptake study in MDA-MB-231 cells treated with PDL1-Dox indicates that PDL-Dox is predominately accumulated in cell surface and unable to reach the nucleus (40× magnified). (**B**) The magnified view of individual cells suggests presence of PDL1-Dox in cell surface (40× magnified). (**C**) Dox is non-specifically accumulated in the nucleus (40× magnified). (**D**) Magnified view suggests the colocalization of Dox with Hoechst dye (as indicated by arrow) (40× magnified). (**E**) The disruption of MDA-MB-231 tumor spheroid in PDL-1-Dox treatment supports the notion that PDL1-Dox can be a potential therapeutic for tumor environment disruption in preclinical tumor model. Arrows indicate the disruption of spheroid in PDL1-Dox treatment (*n* = 3). (**F**) Significant increase in IFN-γ production (pg/mL) in culture media treated with PDL1-Dox using coculture of MDA-MB-231 and activated RAW 264.5 cells as compared to Dox treatment is seen. * *p* < 0.05 (*n* = 4 independent experiment) and results are presented as STDEV in excel.

The data in Figure 4A,B clearly indicate that PDL1-Dox is unable to reach the nucleus of the MDA-MB-231 cells and thus localizes predominantly on the cell surface. This is in contrast to Dox treated cells in Figure 4C,D showing its non-specific accumulation in the nucleus. PDL1-Dox specifically binds to PDL1 receptor and the complex remains mainly on the surface of the cells 24. The presence of tumor stroma is a major barrier for any anti-tumor therapeutic as well as for PDL1 AB. In order to determine the efficacy of the tumor environment disruption of PDL1-Dox, we treated the MDA-MB-231 3D-spheroid culture with 2.5 µM PDL1-Dox, Dox or left it untreated (UT). The data from Figure 4E shows that PDL1-Dox is more effective in disrupting the tumor spheroid compared to Dox. This data resembles the observation in Figure 3A and indicates that the development of PDL1-Dox is a worthwhile approach for the disruption of tumor environment. Furthermore, to evaluate the activation of T-cells in PDL1-Dox treatment, we measured the production of IFN-γ, CD8+ T cell activation cytokine that is released during innate and adaptive immune responses, and its inhibition of the PD-1 stimulatory mechanism. From Figure 4F, it can be seen that the IFN-γ production in PDL1-Dox treatment is significantly higher compared to Dox in a co-cultured condition of MDA-MB-231 and activated RAW 264.7 cells. Literature reports indicate that activation of Raw 264.7 (macrophage) cells with lipopolysaccharide (LPS) can significantly upregulate the PD-1 expression [25,26]. Towards this end, we have utilized the LPS activated Raw 265.7 cells co-cultured with MDA-MB-231 and found the up-modulation of IFN-γ, suggesting the PDL1-Dox mediated inhibition of PD1 and PDL1 interaction. Thus, PDL1-Dox is compatible with the mechanism of ligand association, like the PDL1 AB antibody, and is effective in inducing the synergistic effect of destabilizing tumor spheroid formation and up-modulation of immune cell activation. The rationale of co-culturing the PD1 triggered macrophages with PDL-1 overexpressing MDA-MB-231 [6] would mimic the PD-1 and PDL-1 interaction model in cell culture condition. In this Raw-264.7 and MDA-MB231 co-cultured flask, treatment of PDL1-Dox can inhibit the PD-1 and PDL-1 interaction, resulting activation of macrophages and thus significant upregulation of tumor suppressing pro-inflammatory cytokine, such as IFN-γ.

### 3.4. Imaging

**Figure 5:**
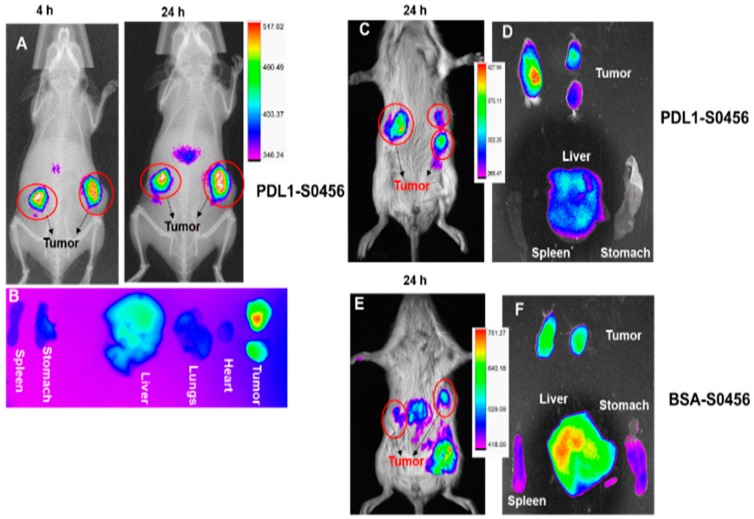
(**A**) NIR imaging of PDL1-S0456 in NSCLC PDx model indicates selective accumulation and retention of dye in tumor mass. (**B**) Higher accumulation of dye in tumor core as compared to other organs support tumor selectivity of the PDL1-S0456 formulation. (**C**,**D**) Higher tumor uptake compared to liver and spleen in TNBC PDX supports PDL1-S0456 as a smart diagnostic tracer for multiple tumor imaging and targeted therapy. (**E**,**F**) indicates high non-specific liver accumulation of non-targeted serum albumin-dye conjugate as compared to tumor (*n* = 2 independent experiment).

With the selective anticancer effect and significant immune activation of PDL1-Dox at the cellular level, we performed near infrared (NIR) optical imaging in TNBC and NSCLC patient derived tumor xenograft (PDx) model with ATZ-conjugated NIR dye, PDL1-S0456. In this regard, we chose PDx models because it generates tumors with features that very closely mimic a human tumor microenvironment that is most ideal for future clinical translation. The rationale of performing NIR-imaging with PDL1-S0456 is due to its significant advantage as a (i) tumor image guided surgery tool in the clinic, and to (ii) understanding tumor selective delivery, tumor retention, and safety to predict therapeutic outcome in different tumor models. The results from Figure 5A,B clearly indicate the selective accumulation and tumor core penetration of PDL1-S0456. The sustained NIR intensity at 4 h and 24 h in NSCLC tumor as shown in Figure 5A indicates the retention of PDL1-S0456, suggesting tumor specificity. The biodistribution in Figure 5B confirms the tumor selectivity of PDL1-S0456 with low non-specific accumulation in liver and spleen. Similarly, PDL1-S0456 is selectively delivered to the TNBC PDx tumor and shows tumor specific delivery and favorable biodistribution as shown in Figure 5C,D. The bovine serum albumin (BSA) conjugated S0456, BSA-S0456 control showed poor specificity to tumor and majority of the dye is accumulated in the liver as compared to the tumor. This data indicates the longer retention and selectivity of PDL1-S0456 in tumors needed for achieving a tumor diagnosis.

## 4. Discussion

Extensive research is ongoing to improve therapeutic outcome ADCs that can enhance their targetability and therapeutic efficacy against tumors. The majority of ADC are used to target the extracellular receptor of cancer cells, followed by receptor mediated-internalization of ADC in cytosol and delivery of payload in endosome [1,27]. Utilizing this mechanism, one can only achieve chemotherapeutic benefits against cancer. In contrast, our approach was to use PDL-1 antibody inhibitor in PDL1-Dox formulation that will bind to the surface of cancer cell and selectively delivery both PDL-1 inhibitor and Dox, resulting a synergistic outcome of chemotherapeutic and immunostimulatory effects. The extensive research majority (two out of three) of clinically approved ADCs are used blood cancer with limited benefits in solid tumor [28]. The phase I/II clinical study of anti-CD74 antibody-doxorubicin conjugate, (IMMU-110) was developed for multiple myeloma [29]. These limitations and challenges of current ADCs technology warranted us to develop PDL1-Dox conjugate for achieving the dual chemo and immunotherapeutic benefits against the solid tumor. In the clinical setting, several antibody-NIR imaging agents have shown an excellent ability to distinguish the tumor lesion from healthy tissue during image-guided surgery as noted in NCT01987375 and NCT01508572 [30] for targeting extracellularly overexpressed VEGF and EGFR receptor. With this note, PDL-1 is an excellent target for developing antibody-NIR or a radio imaging agent that can be utilized for multiple cancer diagnosis and for understanding the cross-talk between cancer cell and T-cell in immune evasion. The up modulation of tumor suppressing pro-inflammatory cytokines, such as IFN-γ in PDL1-Dox treatment supports PDL1-Dox mediated anti-tumor immune cell activation [31]. Furthermore, PDL1-S0456 can be engineered with potent drugs to obtain antibody-dye-drug conjugate for the multimodal image guided therapy and immune modulation. Towards this end, our first approach in developing immune checkpoint antibody inhibitor-drug conjugate and imaging agent demonstrates a rational platform for chemo-guided immunotherapy that can be further developed for other types of cancer.

## 5. Conclusions

In this study we have demonstrated the development of PDL1 antibody drug conjugate to improve the antitumor efficacy of current treatment. The PDL1-Dox treatment has multimodal anticancer effects including tumor acidic pH responsive drug release, apoptosis mediated cancer cell death, targetability of PDL1 receptor and tumor 3D-spheroid disruption, and upmodulating of tumor suppressing IFN-γ mediated immune cell activation. The PDL1-S0456 tool has demonstrated the selective tumor targeting ability in a patient derived tumor model, which is a positive step towards further developing tumor NIR imaging tool for imaging guided surgery of PDL-1 positive tumor in a clinical set up.
